# Squamous cell carcinoma initially occurring on the tongue dorsum: a case series report with molecular analysis

**DOI:** 10.1186/s13000-024-01487-0

**Published:** 2024-04-22

**Authors:** Sawako Ono, Katsutoshi Hirose, Shintaro Sukegawa, Kyoichi Obata, Masanori Masui, Kazuaki Hasegawa, Ai Fujimura, Katsumitsu Shimada, Satoko Nakamura, Akari Teramoto, Yumiko Hori, Eiichi Morii, Daisuke Motooka, Takuro Igawa, Takehiro Tanaka, Hitoshi Nagatsuka, Satoru Toyosawa, Hidetaka Yamamoto

**Affiliations:** 1 Department of Pathology and Oncology, Okayama University Graduate School of Medicine, Dentistry and Pharmaceutical Sciences, 2-5-1 Shikata-cho, Kita-ku, Okayama, Okayama 700-8558 Japan; 2 Department of Oral and Maxillofacial Pathology, Osaka University Graduate School of Dentistry, 1-8 Yamadaoka, Suita, Osaka 565-0871 Japan; 3 Department of Oral and Maxillofacial Surgery, Kagawa University Faculty of Medicine, 1750-1 Ikenobe, Miki-cho, Kita-gun, Kagawa 761-0793 Japan; 4 Department of Oral and Maxillofacial Surgery, Okayama University Graduate School of Medicine, Dentistry and Pharmaceutical Sciences, 2-5-1 Shikata-cho, Kita-ku, Okayama, Okayama 700-8558 Japan; 5 Department of Oral and Maxillofacial Surgery, Kagawa Prefectural Central Hospital, 1-2-1 Asahimachi, Takamatsu, Kagawa 760-8557 Japan; 6 Department of Clinical Pathophysiology, Matsumoto Dental University Graduate School of Oral Medicine, 1780 Gobara Hirooka, Shiojiri, Nagano 399-0781 Japan; 7 Department of Pathology, Kagawa Prefectural Central Hospital, 1-2-1 Asahimachi, Takamatsu, Kagawa 760-8557 Japan; 8 Department of Pathology, Osaka University Graduate School of Medicine, 2-2 Yamadaoka, Suita, Osaka 565-0871 Japan; 9 Genome Information Research Center, Research Institute for Microbial Diseases, Osaka University, 3-1 Yamadaoka, Suita, Osaka 565-0871 Japan; 10 Department of Oral Pathology and Medicine, Okayama University Graduate School of Medicine, Dentistry and Pharmaceutical Sciences, 2-5-1 Shikata-cho, Kita-ku, Okayama, Okayama 700-8558 Japan; 11 Department of Central Laboratory and Surgical Pathology, NHO Osaka National Hospital, 2-1-14 Hoenzaka, Chuo-ku, Osaka, Osaka 540-0006 Japan

**Keywords:** Squamous cell carcinoma, Oral squamous cell carcinoma, Dorsum of the tongue, *TP53*, p53, Next-generation sequencing

## Abstract

**Background:**

Squamous cell carcinoma (SCC) of the dorsum of the tongue is extremely rare, and it clinically resembles various benign lesions. Somatic mutations in *TP53* and some driver genes were implicated in the development of SCC; however, the somatic genetic characteristics of dorsal tongue SCC remain unknown. With a detailed analysis of gene mutations in dorsal tongue SCC, we aimed to better understand its biology.

**Methods:**

Four cases of SCC initially occurring on the tongue dorsum were evaluated for clinical and histological findings and immunohistochemical expression of p53 and p16. Gene mutations were analyzed using next-generation sequencing with a custom panel of driver genes.

**Results:**

We retrospectively investigated 557 cases of tongue SCC, and only four cases of SCC initially occurred on the tongue dorsum. The four patients (cases 1–4) were one woman and three men with a mean age of 53.75 years (range: 15–74 years). Histological analysis revealed well-differentiated SCC. Through molecular analysis, we identified pathogenic somatic mutations, namely, *TP53* p.C176F (c.527G > T) in case 3 and *TP53* p.R282W (c.844 C > T) in case 4. No pathogenic variants were identified in the PI3K/AKT or RAS/RAF pathways. The p53 immunohistochemical examination revealed a wild-type expression pattern in cases 1–3 and strong expression in case 4. The results of p16 immunostaining were negative in all cases.

**Conclusions:**

We described four previously unreported genetic characteristics of dorsal tongue SCC. Somatic *TP53* mutations may contribute to the development of a subset of dorsal tongue SCC; however, more cases with genetic analysis need to be accumulated.

## Introduction

Squamous cell carcinoma (SCC) of the tongue is the most common oral cancer and usually occurs on the lateral border of the tongue [1–3]. SCC occurring on the dorsum of the tongue is extremely rare, accounting for 0–5% of all tongue SCC [2–5]. To the best of our knowledge, only 28 cases of dorsal tongue SCC, including the present four cases, have been described in the English literature [4–21]. These previous cases included only two case series: one with five cases [4] and one with three cases [5]. Other cases have been single-case reports [6–21]. In addition to its rarity, clinical diagnosis of dorsal tongue SCC is challenging as it may resemble various benign lesions; amyloidosis, squamous papilloma, granular cell tumor, lichen planus, median rhomboid glossitis, and chronic candidiasis [3–11, 14, 20]. Owing to the site characteristics, the resection range for dorsal tongue SCC tends to be wider than that for lateral border SCC [18, 20]. Delayed diagnosis of SCC can lead to tongue dysfunction, such as dysarthria and dysphagia [3, 4, 17, 18, 21]. Therefore, it is crucial to arrive at a definitive diagnosis by promptly performing a biopsy when there is a possibility of malignancy [3, 5, 14]. However, dorsal tongue SCC typically exhibits well-differentiated morphology and can be difficult to diagnose as a malignant lesion through small biopsy specimens [3, 20]. As a result, lesions on the dorsum of the tongue are often neglected as they are considered benign [8, 9, 11, 14, 18, 20].

In head and neck SCC (HNSCC), including oral SCC (OSCC), most genetic mutations are associated with tumor suppressor genes such as *TP53*, followed by the phosphatidylinositol 3-kinase (PI3K)/AKT and RAS/RAF pathways [1, 22–27]. Mutations in these genes are closely related to tumorigenesis and prognosis of various malignancies [22–29]. However, there are no reports identifying somatic gene mutations in patients with dorsal tongue SCC, and the genetic characteristics associated with SCC initially occurring on the tongue dorsum remain unknown. Inherited mutations in *TP53* encoding transcription factor p53 increase the risk of various malignancies with early onset [30, 31]. Yamasaki et al. reported a case of dorsal tongue SCC with a germline *TP53* mutation (p.R280*, c.838 A > T) [19]: the patient had multiple cancers associated with germline *TP53* mutations, including esophageal, gastric, and renal cancers (Table 1). These results suggested that *TP53* mutations are involved in the development of dorsal tongue SCC.

**Table 1:** Summary of characteristics of dorsal tongue SCC in previous reported cases and the present series

	Age (y)/ Sex	Medical history	Tobacco history	Histology	TP53 mutations	p53 IHC
Yamasaki et al. [19]	69/M	Esophageal, gastric, and renal cancer	Former smoker	Well-differentiated SCC	Germline p.R280*	Null
Abe et al. [20]	69/F	No	No	Well-differentiated SCC	NA	Positive
Case 1	63/F	No	Former smoker 40 pack-years	Well-differentiated SCC	No	WT
Case 2	74/M	Urothelial cancer	Current smoker 5 pack-years or more	Well-differentiated SCC	No	WT
Case 3	63/M	MDS	No	Verrucous carcinoma with dysplasia or minimal invasion (well-differentiated SCC)	Somatic p.C176F	WT
Case 4	15/M	IBMFS	No	Well-differentiated SCC	Somatic p.R282W	Positive

SCC, squamous cell carcinoma; IHC, immunohistochemical staining; M, male; F, female; NA, not available; WT, wild-type; MDS, myelodysplastic syndrome; IBMFS, inherited bone marrow failure syndrome

A detailed analysis of gene mutations in dorsal tongue SCC could enhance our understanding of its biology. Here, we performed clinical, pathological, and genetic analyses of a case series of dorsal tongue SCC.

## Methods

### Patient selection

We retrospectively investigated 557 cases of tongue SCC obtained from the pathology files of Okayama University Hospital, Osaka University Dental Hospital, Kagawa University Hospital, and Kagawa Prefectural Central Hospital. Among SCC cases occurring on the tongue dorsum, we excluded cases of non-primary SCC and simultaneous SCC at the tongue dorsum and other sites. The final diagnosis of dorsal tongue SCC was made by two pathologists (SO and KH). Formalin-fixed, paraffin-embedded (FFPE) tissues were retrieved for four patients with dorsal tongue SCC (cases 1–4). This study was approved by the Ethical Review Board of the Graduate School of Dentistry, Osaka University (No. R5-E11).

### Histological and immunohistochemical examination

Resected tissue samples were fixed with 10% formalin, embedded in paraffin, cut into 4-µm-thick serial sections, and used for hematoxylin and eosin (H&E) and immunohistochemical staining. TNM classification was performed according to the 8th edition of the Union for International Cancer Control. Immunohistochemical staining with a primary antibody against p53 (clone-DO7) and p16 (clone-E6H4) was performed using a Roche Ventana BenchMark GX autostainer (Ventana Medical Systems, Tucson, AZ, USA) according to the manufacturer’s instructions.

### Molecular analysis

To examine mutational status, we performed next-generation sequencing (NGS) with a custom panel, as previously described [32]. The gene panel was designed using SureDesign (https://earray.chem.agilent.com/suredesign) to cover the exons of *TP53* or genes associated with the PI3K/AKT and RAS/RAF signaling pathways (*PIK3CA*, *AKT1*, *PTEN*, *BRAF*, *MAP3K3*, *KRAS*, *NRAS*, *HRAS*, and *RASA1*) [32, 33]. Two pathologists (SO and KH) identified FFPE blocks with more than 15% abnormal tissue content. Genomic DNA was extracted from FFPE tissues using the QIAamp DNA FFPE Tissue Kit (Qiagen, Valencia, CA, USA) according to the manufacturer’s instructions. On average, 70 ng of the extracted DNA was fragmented into 150–200 bp using SureSelect Fragmentation Enzyme (Agilent Technologies, Inc., Santa Clara, CA, USA). Sequence libraries were prepared using the custom SureSelect Low Input Target Enrichment System (Agilent Technologies, Inc., Santa Clara, CA, USA) according to the manufacturer’s instructions and sequenced with Illumina MiSeq (Illumina, Santa Clara, CA, USA) according to the manufacturer’s instructions. SureCall ver4.2 (https://www.agilent.com/en/download-software-surecall) was used for variant calling [34]. DNA from introns or noncoding DNA was excluded.

## Results

### Clinical findings

#### Case 1

**Fig. 1:**
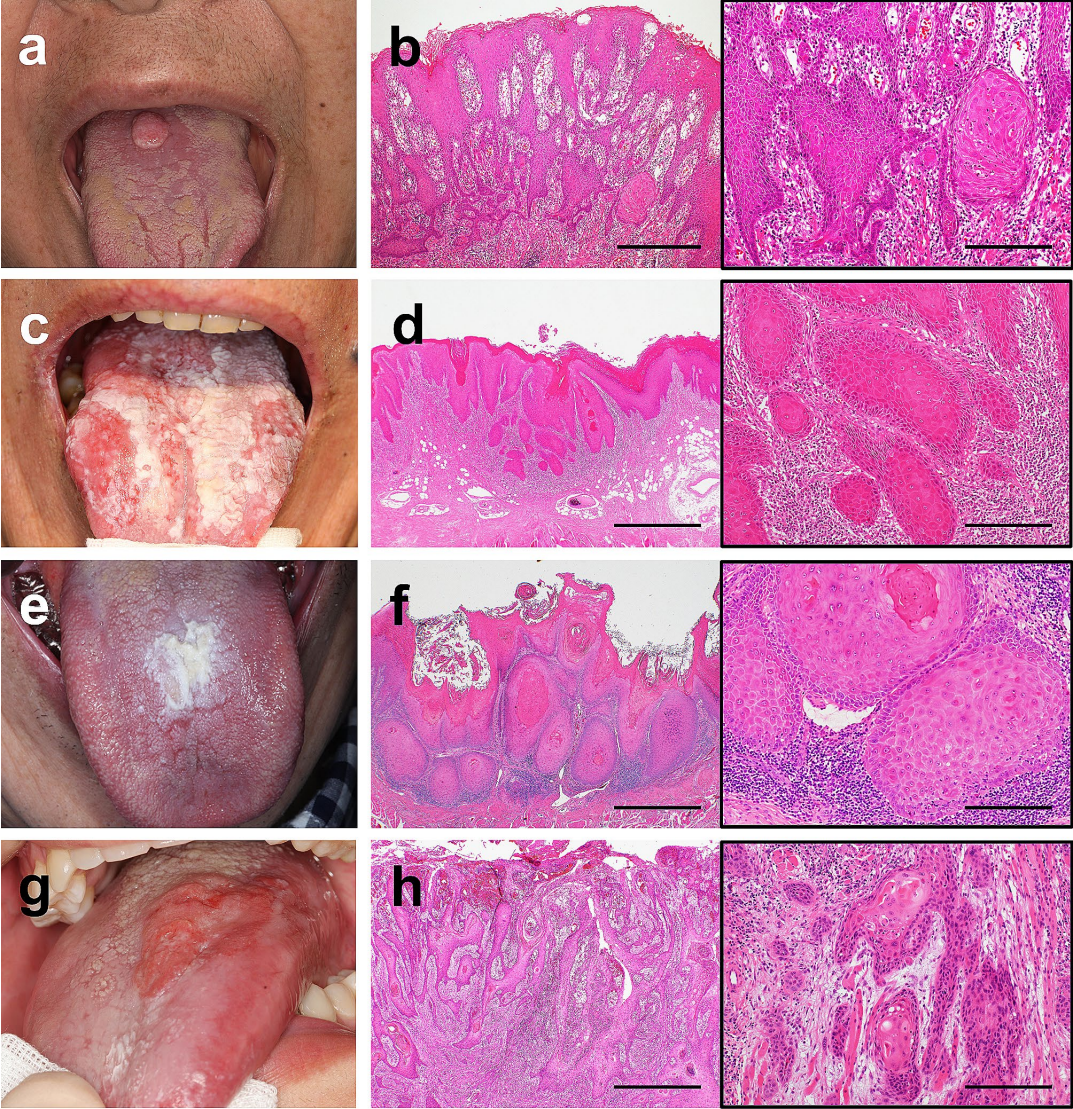
Intraoral and histological findings of dorsal tongue SCC. Intraoral findings of cases 1 (**a**), 2 (**c**), 3 (**e**), and 4 (**g**). Representative histological findings for cases 1 (**b**), 2 (**d**), 3 (**f**), and 4 (**h**). Black boxes show higher magnifications of **b**, **d**, **f**, **h**. The tumors exhibited irregular epithelial stratification and keratin pearl formation within the ridges. The tumors also exhibited nests or islands and infiltrated connective tissues. SCC, squamous cell carcinoma. Scale bars: 500 μm in Fig. **b**; 1000 μm in Figs. **d**, **f** and **h**; 200 μm in black boxes

A 63-year-old female patient complained of discomfort in the dorsum of the tongue. She had no history or family history of cancer. She was a smoker and did not consume alcohol. Intraoral examination revealed a well-circumscribed, elastic, soft mass measuring 13 × 9 mm in the midline to the right side of the tongue dorsum (Fig. 1a). The patient underwent surgical resection under general anesthesia. The tumor grade was T1N0M0 (stage I). No signs of recurrence or metastasis were observed at the final follow-up 72 months later.

#### Case 2

A 74-year-old male patient was followed up for dorsal tongue oral leukoplakia (OL) for 10 years. He had a history of bladder and ureteral cancer (urothelial carcinoma) but no family history of cancer. He was a smoker and did not consume alcohol. An intraoral examination revealed an irregular, white, flat lesion on the dorsal surface of the tongue (Fig. 1c). The patient underwent surgical resection under general anesthesia. The tumor grade was T1N0M0 (stage I). No signs of recurrence or metastasis were observed at the final follow-up visit 60 months later.

#### Case 3

A 63-year-old male patient was followed-up for dorsal tongue OL for 2 years. He had a history of myelodysplastic syndrome (MDS). He had undergone hematopoietic stem cell transplantation (HSCT) for MDS 11 years prior and developed chronic graft-versus-host disease in the gastrointestinal tract one year after HSCT. He had no history of smoking or alcohol consumption. Intraoral examination revealed a white, slightly elevated lesion with an irregular surface measuring 17 × 9 mm at the midline of the dorsum of the tongue (Fig. 1e). The patient underwent surgical resection under general anesthesia. The tumor grade was T1N0M0 (stage I). No signs of recurrence or metastasis were observed at the last follow-up visit 13 months later.

#### Case 4

A 15-year-old boy was followed-up for dorsal tongue OL. The patient had a history of inherited bone marrow failure syndrome (IBMFS). He had no history of smoking or alcohol consumption. Intraoral examination revealed an erythematous, slightly elevated lesion measuring 30 × 15 mm in the midline to the left side of the tongue dorsum (Fig. 1g). The patient underwent surgical resection under general anesthesia. The tumor grade was T2N0M0 (stage II). SCC recurred 52 months after resection, and the patient died of immunodeficiency due to IBMFS 104 months after resection.

### Histological findings

Histological examination revealed the proliferation of neoplastic squamous epithelial cells with various levels of cytological atypia (Fig. 1b, d, f, and h). The tumors exhibited irregular epithelial stratification and keratin pearl formation within the ridges. In cases 1, 2 & 4, tumors exhibiting nest or island structures infiltrated connective tissues, and we diagnosed well-differentiated SCC (Fig. 1b, d, and h). In case 3, the tumor showed a warty keratinized surface and pushing infiltration pattern with mild cytological atypia. The histological findings in case 3 were consistent with verrucous carcinoma (VC), which is a subtype of SCC (Fig. 1f). Moreover, coexistence of foci of well-differentiated conventional SCC with VC was observed in the deep area (Fig. 1f, black box). The conventional SCC components showed irregular nests of cells with higher nuclear to cytoplasmic ratios and increased size of nucleoli (Fig. 1f, black box). The distance from the deepest point of invasion of the conventional SCC component to the nearest focus of VC was approximately 1000 μm. Thus, we finally diagnosed case 3 as VC with dysplasia or minimal invasion (VCDMI) [35].

There were no findings of vascular or perineural invasion in the specimens of either of the four cases. In addition, there were no findings suggestive of amyloidosis, granular cell tumors, lichen planus, or chronic candidiasis. No SCC lesions were detected at locations other than the dorsum of the tongue.

### Molecular genetic analysis and immunohistochemical findings

**Fig. 2:**
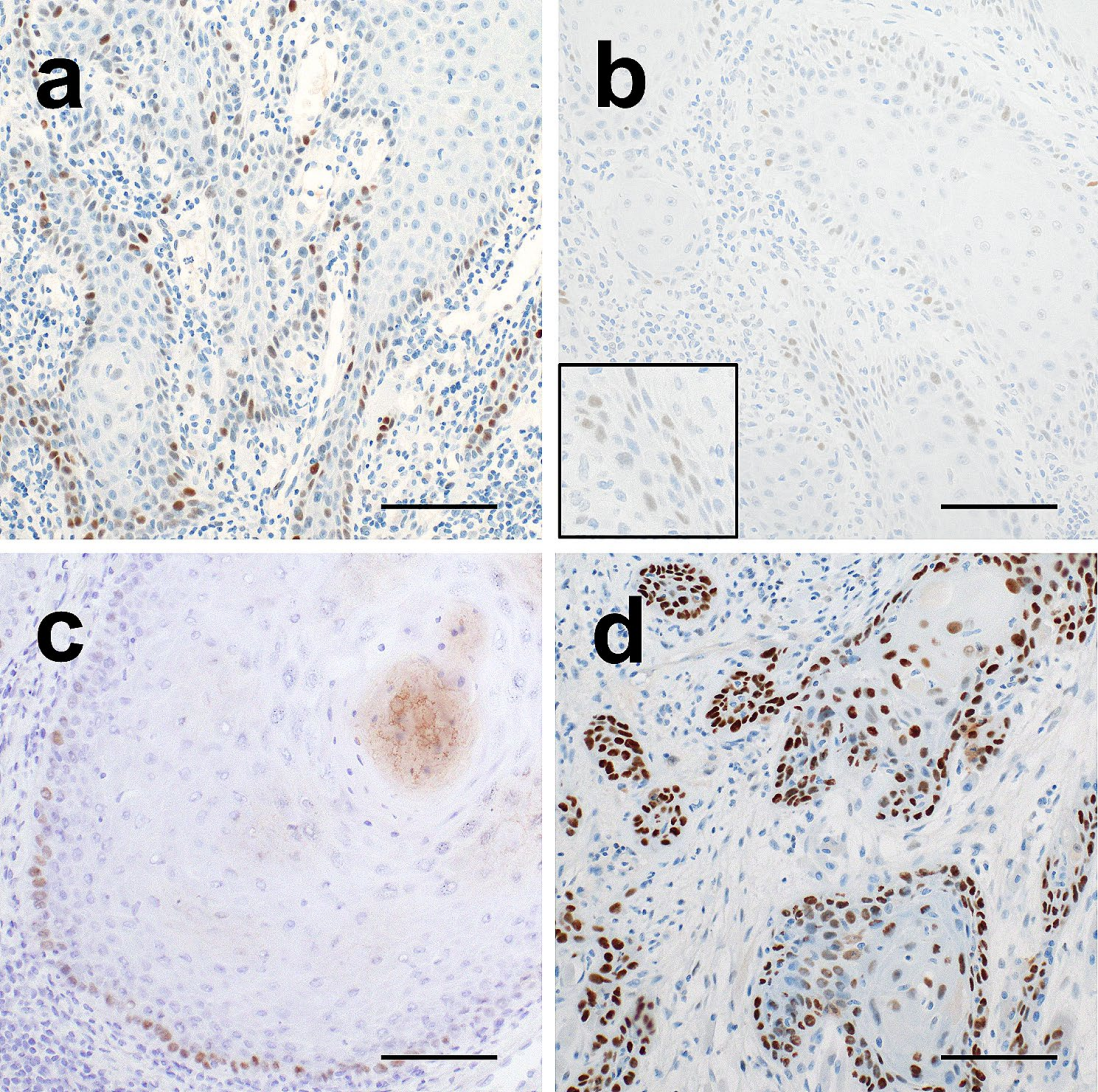
Immunohistochemical analysis of dorsal tongue SCC. Representative immunohistochemical staining of p53 in case 1 (**a**), 2 (**b**), 3 (**c**), and 4 (**d**). Cases 1–3 showed normal wild-type expression pattern (negative to weakly positive), and case 4 showed strong expression. SCC, squamous cell carcinoma. Scale bars: 100 μm in Figs. **a**-**d**

Genetic mutations were evaluated in all cases using NGS with a custom panel, as previously described [32]. NGS identified a *TP53* p.C176F (c.527G > T) somatic mutation classified as pathogenic in case 3 and a *TP53* p.R282W (c.844 C > T) somatic mutation classified as pathogenic in case 4 (Table 1). No pathogenic variants of *PIK3CA*, *AKT1*, *PTEN*, *BRAF*, *MAP3K3*, *KRAS*, *NRAS*, *HRAS*, or *RASA1* were identified. Immunohistochemical examination of p53 revealed a wild-type staining pattern (negative to weakly positive) in cases 1–3 (Fig. 2a to c), and a strong staining pattern in basal and suprabasal layers in case 4 (Fig. 2d). The p16 immunostaining results were negative in all four cases. The results of the genetic and immunohistochemical analyses in previously reported cases and the present series are summarized in Table 1.

## Discussion

We retrospectively investigated 557 cases of tongue SCC, and only four cases of SCC initially occurred on the tongue dorsum. Clinicopathological and genetic characteristics differ depending on the oral anatomical sites of OSCC [36], suggesting that dorsal tongue SCC may have characteristics different from those of SCC occurring at other sites. To the best of our knowledge, this study is the first to report a case series involving the clinical, pathological, and genetic analyses of dorsal tongue SCC.

Several large-scale studies have reported that most tongue cancers occur on the lateral border; cancers occurring on the tongue dorsum are very rare, with an incidence of 2.9–5.1% [37–41]. These large-scale studies of tongue cancers included non-primary SCC cases, multiple SCC cases, cases with histological types other than SCC such as mucoepidermoid carcinoma, and cases without a pathological diagnosis [37–41]. In large-scale studies limited to tongue SCC, the incidence of dorsal tongue SCC was reported to be 0% (0/222 cases [2] and 0/302 cases [3]). In the three case series of dorsal tongue SCC, including the present study, the incidence of SCC initially occurring on the tongue dorsum was 5.1% (5/99 cases) [4], 0.82% (3/368 cases) [5], and 0.72% (4/557 cases), accounting for approximately 1% incidence. The country distribution of reported dorsal tongue SCC was as follows: Japan had the most cases (39.3%, 11/28 cases), followed by Israel (25%, 7/28 cases), and the USA (17.9%, 5/28 cases). Although regional differences might affect the incidence of dorsal tongue SCC, its occurrence remains rare. To summarize the 28 cases of dorsal tongue SCC reported thus far, the mean age of patients was 57.96 years (range: 15–80 years), and the male-to-female ratio was 13:15 [4–21]. In general, most patients with tongue SCC are aged 50–70 years, with the occurrence slightly predominating in males [1, 37, 38, 41]. Histologically, dorsal tongue SCC has been reported to be well or moderately differentiated (75 and 12.5%, respectively), while most tongue SCCs are well or moderately differentiated [1, 41]. Although it is difficult to compare dorsal tongue SCC with tongue SCC, the clinical and pathological findings were not significantly different.

Verrucous carcinoma is a well differentiated non-metastatic SCC subtype that may progress to invasive conventional SCC [1]. Approximately 20% of VC cases of the oral cavity coexist with a conventional SCC component [42]. Patel et al. categorized VC into three types: pure VC, VCDMI, and SCC arising in VC (SCC-VC) [35]. Cases with a distance of less than 2 mm from the deepest point of invasion of the conventional SCC component to the nearest focus of VC are defined as VCDMI, and cases with a distance of 2 mm or more are defined as SCC-VC [35]. In case 3 of the present study, the distance from the deepest point of invasion of the conventional SCC component to the nearest focus of VC was approximately 1000 μm. Thus, we finally diagnosed case 3 as VCDMI. Compared to SCC-VC, VC and VCDMI show good prognosis with a low recurrence rate, either locally or in regional lymph nodes [35]. Consistently, case 3 showed no signs of recurrence or metastasis at the last follow-up visit 13 months later.

Somatic mutations in *TP53* and genes associated with the PI3K/AKT and RAS/RAF signaling pathways have potential roles as drivers of HNSCC and OSCC development [1, 22–27]. Kobayashi et al. reported that the most frequently mutated gene among 284 HNSCC cases was *TP53* (67%), followed by *PIK3CA* (8%), *AKT1* (4%), and *HRAS* (3%) [26]. Similarly, frequently mutated genes in OSCC were *TP53* (65–66%), *PIK3CA* (16.8–20%), *AKT1* (0–14%), and *HRAS* (9–9.3%) [23, 25]. Although no mutations in the PI3K/AKT and RAS/RAF signaling pathways were detected in the present study, we identified somatic *TP53* mutations in 50% of the cases (2/4 cases; Table 1). The results of the genetic analysis in the present study are consistent with the distribution of somatic mutations in HNSCC and OSCC [1, 23, 25, 26]. *TP53* is activated in response to DNA damage and is mutated in most common human malignancies, with various ranges depending on the stage and etiology of the tumors [43]. Both *TP53* p.C176F and p.R282W mutations are in the DNA-binding domain of p53 (Table 1) and have been reported as pathogenic and hotspot mutations in HNSCC [44]. Two previous dorsal tongue SCC cases have been reported with p53 immunohistochemical staining; in one case, a germline *TP53* mutation was identified (Table 1) [19, 20]. Combining these cases with those reported in the present study, the frequency of occurrence of *TP53* mutations was 60% (3/5 cases) and the frequency of mutant p53 immunostaining pattern (null or positive) was 50% (3/6 cases; Table 1). These observations suggest that somatic *TP53* mutations are involved in the development of a subset of SCC of the tongue dorsum.

A limitation of this study is its small sample size owing to the rarity of dorsal tongue SCC. Further studies are required to determine the genetic characteristics of dorsal tongue SCC and the association between gene mutations and SCC biology in a larger cohort of cases.

## Conclusions

In the present study, we describe four cases of previously unreported genetic characteristics of dorsal tongue SCC. Our results suggest that somatic *TP53* mutations may contribute to the development of a subset of dorsal tongue SCC; however, more cases with genetic analysis need to be accumulated.

## Data Availability

The surgical materials and datasets analyzed in this study are available from the corresponding author upon request.

## Abbreviations

AKT1: AKT serine/threonine kinase 1
BRAF: B-RAf proto-oncogene, serine/threonine kinase
FFPE: Formalin-Fixed Paraffin-Embedded
HNSCC: Head and Neck Squamous Cell Carcinoma
HRAS: HRAS proto-oncogene, GTPase
HSCT: Hematopoietic Stem Cell Transplantation
IBMFS: Inherited Bone Marrow Failure Syndrome
KRAS: KRAS proto-oncogene, GTPase
MAP3K3: Mitogen-Activated Protein Kinase 3
NGS: Next-Generation Sequencing
NRAS: NRAS proto-oncogene, GTPase
OL: Oral Leukoplakia
OSCC: Oral Squamous Cell Carcinoma
PI3K: Phosphatidylinositol 3-Kinase
PIK3CA: Phosphatidylinositol-4, 5-Bisphosphate 3-Kinase Catalytic subunit Alpha
PTEN: Phosphatase and Tensin Homolog
RASA1: RAS P21 Protein Activator 1
SCC: Squamous Cell Carcinoma
VC: Verrucous Carcinoma
VCDMI: Verrucous Carcinoma with Dysplasia or Minimal Invasion
